# A Prospective Study on Algorithms Adapted to the Spatial Frequency in Tomography

**DOI:** 10.1155/IJBI/2006/34043

**Published:** 2006-06-04

**Authors:** Vincent Israel-Jost, Philippe Choquet, André Constantinesco

**Affiliations:** ^1^ Institut de Recherche Mathématiques Avancées, 7 rue René Descartes, Université Louis Pasteur, Strasbourg Cedex 67084, France; ^2^ Service de Biophysique et Médecine Nucléaire, Hôpital de Hautepierre, 1 avenue Molière, Strasbourg 67098, France

## Abstract

The use of iterative algorithms in tomographic reconstruction always leads to a frequency adapted rate of convergence in that low frequencies are accurately reconstructed after a few iterations, while high frequencies sometimes require many more computations. In this paper, we propose to build *frequency adapted* (FA) algorithms based on a condition of incomplete backprojection and propose an FA *simultaneous algebraic reconstruction technique* (FA-SART) algorithm as an example. The results obtained with the FA-SART algorithm demonstrate a very fast convergence on a highly detailed phantom when compared to the original SART algorithm. Though the use of such an FA algorithm may seem difficult, we specify in which case it is relevant and propose several ways to improve the reconstruction process with FA algorithms.


Hindawi Publishing Corporation
International Journal of Biomedical Imaging
Volume 2006, Article ID 34043, Pages 1–6
DOI 10.1155/IJBI/2006/34043

### A Prospective Study on Algorithms Adapted to the Spatial Frequency in Tomography

**Vincent Israel-Jost,[1,2] Philippe Choquet,[2] and André Constantinesco[2]**

[1] *Institut de Recherche Mathématiques Avancées, 7 rue René Descartes, Université Louis Pasteur, 67084 Strasbourg Cedex, France*
[2] *Service de Biophysique et Médecine Nucléaire, Hôpital de Hautepierre, 1 avenue Molière, 67098 Strasbourg, France*

Received 1 December 2005; Revised 23 April 2006; Accepted 25 April 2006

The use of iterative algorithms in tomographic reconstruction always leads to a frequency adapted rate of convergence in that low frequencies are accurately reconstructed after a few iterations, while high frequencies sometimes require many more computations. In this paper, we propose to build *frequency adapted* (FA) algorithms based on a condition of incomplete backprojection and propose an FA *simultaneous algebraic reconstruction technique* (FA-SART) algorithm as an example. The results obtained with the FA-SART algorithm demonstrate a very fast convergence on a highly detailed phantom when compared to the original SART algorithm. Though the use of such an FA algorithm may seem difficult, we specify in which case it is relevant and propose several ways to improve the reconstruction process with FA algorithms.

Copyright © 2006 Vincent Israel-Jost et al. This is an open access article distributed under the Creative Commons Attribution License, which permits unrestricted use, distribution, and reproduction in any medium, provided the original work is properly cited.

### 1. INTRODUCTION

Iterative methods are increasingly used in computed tomography (image reconstruction from projections) where in most cases they advantageously replace analytical methods. The latter methods, whose success is based upon a great robustness and speed, unfortunately cannot take into account a certain number of physical and geometrical parameters which should appear in the *point spread function* (PSF). These parameters (intrinsic resolution of the detector, spatial variation of sensitivity, and others, related to specific applications) can be easily incorporated in the projection operator that models the process of imaging in iterative methods. This however does not change the nature of the problem to be solved, formulated as a large system of linear equations:

$$Ax = b, \tag{1}$$

where $A$ is the projection operator, $x$ is typically a set of (unknown) values taken by the voxels in the 3D space (attenuation coefficients, activity ...), and $b$ is the set of measurements, often organized as a set of projection images.

In addition, besides the exponential growth in computer processing power that obviously plays an important role in the feasibility of large computations, solving methods for (1) profit from a broad effort realized in general inverse problems methods and from a continuous exchange between theory and applications.

One important ongoing study on the speed of convergence is contingent on the consideration of different algorithms (*algebraic reconstruction technique* (ART) [1], *simultaneous algebraic reconstruction technique* (SART) [2], *expectation maximization* (EM) [3], or conjugated gradient for the most famous), different types of implementation (sequential (Seq), simultaneous (Sim), or block-iterative (BI)), regularizations, or relaxation parameters (see [4] for details). A solid mathematical framework was formed, which in most cases tendered proof of convergence, including at times for the inconsistent case—generally that of computed tomography.

The idea of the work presented here has risen from the fact that the high frequencies (the details) are always reconstructed after the low frequencies in iterative methods for computed tomography. In the case of highly detailed objects (in a sense that we will later specify), an important number of iterations might be required to obtain the precision expected on the reconstructed image. This very general fact has been experienced by all who worked with iterative algorithms, and keys for the understanding of this phenomena can be found in [5], but in a framework of integral geometry resulting in a Dirac projection PSF and thus quite different from our context.

In this article, we propose a way to accelerate the convergence of iterative methods in the specific case of highly detailed objects, based on the use of an incomplete backprojection operator. This can be roughly formulated as a weighting of each correction $d_{i,j}^k$ of voxel $j$ by equation $i$ during the $k$th iteration with weights $w_{i,j}^k$ such as $\sum_i w_{i,j}^k = 1$ for all $j$ and $k$ and the *incomplete backprojection condition* (IBC):

$$w_{i,j}^k = 0 \quad \text{for some } i, \text{ even if } A_{i,j} \neq 0. \tag{2}$$

To our knowledge, this condition has not been thus far reported in the use of reconstruction algorithms for computed tomography. Besides the formulation of a frequency adapted (FA) algorithm that realizes the reconstruction with IBC starting from the well-known SART algorithm, a major concern of this paper is to explain how the IBC permits to accelerate the convergence by updating the value of the voxels from the most relevant measurements and to give experimental results to support both the convergence of the algorithm and the gain obtained when compared to SART.

In Section 2, we describe the type of problems for which we believe the adaptation involved by the IBC can be useful and formulate the IBC in terms of projections onto convex sets. We explain the IBC for it to realize the expected acceleration and give an adaptation of the simultaneous algebraic reconstruction technique (SART) algorithm of Andersen and Kak [2] modified by the IBC. In Section 3, we show results on simulations, phantom, and small animal with a comparison between SART and FA-SART. Section 4 is a discussion on the proposed method which also suggests a number of different use for FA algorithms and Section 5 concludes the paper.

### 2. BUILDING AN ALGORITHM ADAPTED TO THE SPATIAL FREQUENCY

In computed tomography like in other applications leading to inverse problems, a set of discrete measurements is physically obtained, presumably by the application of some linear operator $A$ to a studied object, discretized into a set of unknowns. For each unknown $x_j$ (a voxel in tomography), the point spread function (PSF) (we should actually talk about the voxel spread function) appears in the column $A_{\bullet,j}$ of the matrix $A$ whose coefficients are assumed to be nonnegative here, to simplify the notations.

For some imaging techniques, the paradigmatic case of which is single photon emission computed tomography (SPECT), this PSF is always different from a Dirac function since it notably includes the detector components (collimator, scintillator, photo-multiplicators, electronic boards, etc.). Indeed, the photons emitted from a voxel $j$ reach far more than one single pixel on the detector and one has to take this into account in the matrix $A$ when modeling this problem. PSF in SPECT is generally modelled by discretized Gaussian functions but some authors proposed more elaborate models, especially for pinhole SPECT (see Metzler et al. [6]).

But whatever the chosen model is, the actual situation is that two close voxels have many measurements in common in their PSF (see Figure 1). This may result in a slow rate of convergence: the backprojection of these shared measurements leads to a smoothing for the voxels' values, that only tends to vanish as the number of iterations increases. This is not necessarily inadequate since when a large and homogeneous structure is to be imaged, this smoothing will mainly reduce the noise if one stops the iterations before the noise is reconstructed (Figure 2(a)). But when considering an image of projection with high frequencies, using these shared measurements in the backprojection, or at least the furthest away from the center of the PSF, will hold the voxels' values distant from the solution $x^*$ for a certain number of iterations (Figure 2(b)). Throughout this paper, we will characterize higly detailed objects in an informal manner by reference to this figure, that is, when significant details of the image are of the same size or smaller than the PSF's width.

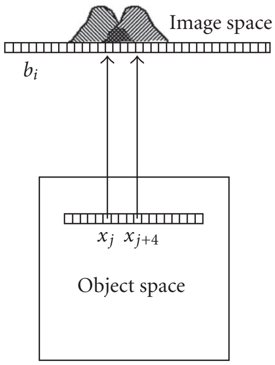

FIGURE 1: Overlapping between two close voxels' PSF. The darkest zone will be used to reconstruct voxel $x_j$ as well as $x_{j+4}$ (and all those in between), thus leading to a smoothed reconstruction after only a few iterations.

Our idea is to only use the most central measurements of the PSF to accelerate the convergence in the case of imaging with high spatial frequencies. To do so, we considered the SART algorithm whose iteration is

$$x_j^{k+1} = x_j^k + \frac{\lambda}{\sum_{i=1}^M A_{i,j}} \sum_{i=1}^M \frac{b_i - (Ax^k)_i}{\sum_{j=1}^N A_{i,j}} A_{i,j}, \tag{3}$$

where $j = 1, \ldots, N$ indexes the voxels and $i = 1, \ldots, M$ indexes the pixels so that $x^k \in \mathbb{R}^N$ for all $k, b \in \mathbb{R}^M$, $A$ is an MxN matrix modelling the problem and $\lambda$ is the relaxation parameter. This can also be written in a matrix form discussed by Jiang and Wang [7] ($T$ denotes the transpose of a matrix or a vector):

$$x^{(k+1)} = x^{(k)} + \lambda V A^T W (b - Ax^{(k)}), \tag{4}$$

with $V = \text{diag}(1/\sum_{i=1}^M A_{i,j})$ an NxN diagonal matrix and $W = \text{diag}(1/\sum_{j=1}^N A_{i,j})$ an MxM diagonal matrix. We also assume the conditions $\sum_{i=1}^M A_{i,j} \neq 0$ for all $j$ and $\sum_{j=1}^N A_{i,j} \neq 0$ for all $i$.

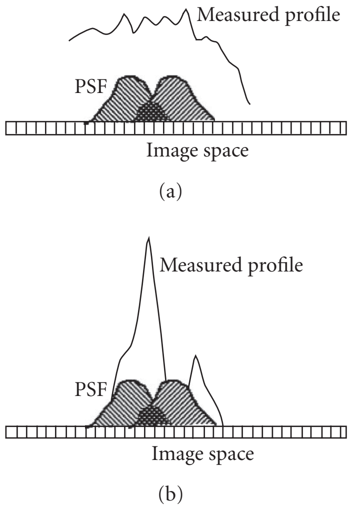

Figure 2: Comparison of the two main cases, when a profile corresponding to a large and homogeneous structure is measured with noise (a), and when the measured profile corresponds to a higly detailed object (b). On (a), using all the discrete measurements of voxel's PSF will smooth the reconstruction which will reduce the noise. On (b), "important" high frequencies are smoothed and more iterations will be needed to achieve an accurate reconstruction.

Although this might sound antinomic with the *simultaneous* of SART, we can write a component Seq form of SART

$$x_j^{k+1} = x_j^k + \lambda \frac{b_i - \langle a^i, x^k \rangle}{\|a^i\|_1} \mathbf{1}_{a_j^i > 0} \tag{5}$$

that can be derived from the analysis presented in [8] by Censor and Elfving. Then, applying this algorithm in the matrix form of (4), it is well known that the new estimate $x^{k+1}$ is the projection of $x^k$ onto the hyperplane $H_i = \{x \in \mathbb{R}^N / \langle a^i, x \rangle = b_i\}$ if $\lambda = 1$.

This is our starting point for a frequency adapted algorithm based on SART. For the purpose of the incomplete backprojection condition, given $0 \le \rho \le 1$, let $A^\rho$ denote the matrix

$$A^\rho := \begin{cases} A_{i,j} & \text{if } A_{i,j} \ge \rho \cdot \|A_{\bullet,j}\|_\infty, \\ 0 & \text{otherwise,} \end{cases} \tag{6}$$

so that $A^\rho$ only keeps in each column the coefficients of $A$ higher than $\rho$ times the top of the PSF. We also define the matrix $V^\rho := \operatorname{diag}(1/\sum_{i=1}^M A_{i,j}^\rho)$ and $W^\rho := \operatorname{diag}(1/\sum_{j=1}^N A_{i,j}^\rho)$. Then, following (4) we define the next iterate corresponding to the treatement of a single projection $i$ as

$$x^{(k+1)} = x^{(k)} + \lambda V^\rho A^{\rho T} W^\rho (b - Ax^{(k)}) = P_i^\rho (x^k). \tag{7}$$

It can been showen in the exact same manner as for (4) (in the sequential case) that $P_i^\rho(x)$ projects $x$ onto the hyperplane $H_i$. But (7) is also designed to have $P_i^\rho(x)$ in the affine subspace of $\mathbb{R}^N$:

$$\Omega_i^k = \left\{ x \in \mathbb{R}^N / P_i^\rho(x)_j = x_j \ \forall j / A_{i,j} < \rho \cdot \|A_{\bullet,j}\|_\infty \right\} \tag{8}$$

thus projecting the current estimate onto $C_i^k := H_i \cap \Omega_i^k$, which is a closed convex set of $\mathbb{R}^N$.

Now, we want to consider the scheme (7) in its natural case, that is, simultaneous. Thus we change the iteration (3) for the following.

*Algorithm 2.1* (FA-SART).

$$x_j^{k+1} = x_j^k + \frac{\lambda}{\sum_{i=1}^M A_{i,j}^\rho} \sum_{i=1}^M \frac{b_i - (Ax^k)_i}{\sum_{j=1}^N A_{i,j}^\rho} A_{i,j}^\rho. \tag{9}$$

The BI version requires some sophistication in the notations and for this purpose, we adopt pretty much the same ones as Censor and Elfving in [8]. Let then $T$ be the number of blocks and, for $t = 1, 2, \ldots, T$, let the blocks of indices $B_t \subseteq \{1, 2, \ldots, M\}$ be an ordered subset of the form $B_t = \{l_1^t, l_2^t, \ldots, l_{M(t)}^t\}$, where $M(t)$ is the number of elements in $B_t$. In our case, we define $T = M$ and for each $t, B_t = \{t\}$. For $t = 1, 2, \ldots, T$, let $A_t$ denote the matrix formed by taking all the rows $\{a^i\}$ of $A$ whose indices belong to the block of indices $B_t$, that is, $A_t := a^t$. The same applies to $A^\rho$ with $A_t^\rho$ the matrix formed by taking all the rows $\{\tilde{a}^i\}$ of $A^\rho$ whose indices belong to the block of indices $B_t$ and $\tilde{a}_c^{j,t(k)}$ the $j$th column of $A_t^\rho$.

The vector $b$ is partitioned similarly with $b^t$ denoting the elements of $b$ whose indices belong to the block of indices $B_t$, that is, $b^t := b_t$. Let us also denote $a_c^{j,t(k)}$ the $j$th column of $A_t$, then the BI FA-SART writes as follows.

*Algorithm 2.2* (BI FA-SART).

$$x_j^{k+1} = x_j^k + \frac{\lambda}{\|\tilde{a}_c^{j,t(k)}\|_1} \sum_{i=1}^{M(t(k))} \frac{b_i^{t(k)} - \langle a^{l_i^{t(k)}}, x^k \rangle}{\|\tilde{a}^{l_i^{t(k)}}\|_1} \tilde{a}_j^{l_i^{t(k)}}. \tag{10}$$

These algorithms realize a backprojection characterized by a contracted cone compared to the original SART (see Figure 3).

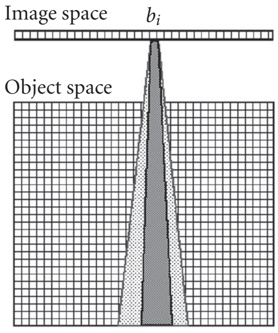

Figure 3: The original backprojection cone corresponding to a pixel $b_i$ and composed of voxels $v_j$, such as $A_{i,j} > 0$ (light grey), is replaced by a set of voxels, such as $A_{i,j} > \rho \cdot \|A_{\bullet,j}\|_\infty$ (dark grey).

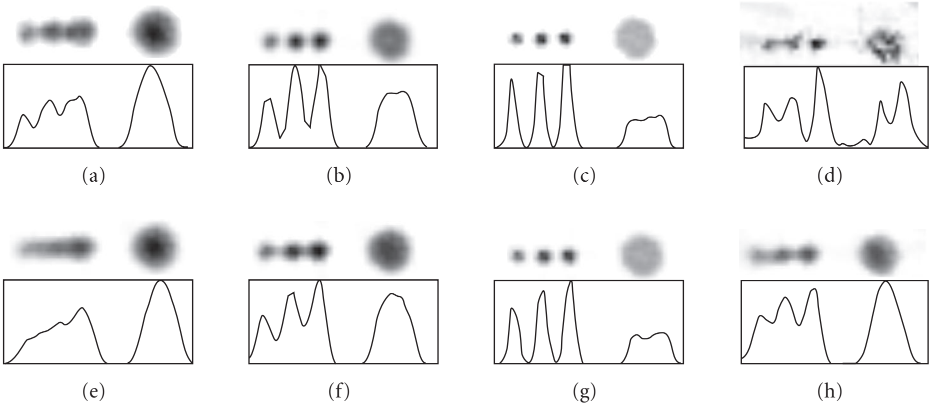

Figure 4: Simulation with a computed phantom composed of three small spots and a bigger structure. (a)–(d) show results obtained with the FA-SART algorithm, $\rho = 1$, and (e)–(h) show results with the SART algorithm (which is FA-SART with $\rho = 0$). The pinhole aperture was 2.5 mm for (a) and (e), 1.5 mm for (b) and (f), 0.025 mm for (c) and (g), and 1.5 mm with noise added onto the projections for (d) and (h). These results show improvements with FA-SART when the PSF is large (2.5 mm and 1.5 mm) and better images with SART when the data are noisy (Gaussian noise, 20% of the maximum of the data), especially for big structures. No significant difference is visible when the PSF is small, because the stage of deconvolution is of less importance during the reconstruction process.

### 3. RESULTS

### 3.1. Material and method

The applications shown here are simulations and pinhole SPECT acquisitions made on a small animal single head dedicated SPECT gamma camera (Gaede Medizinsysteme GMBH, Freiburg, Germany) with a 6.5 mm NaI(Tl) crystal 25 photomultipliers and a small field of view of 17 cm $\times$ 17 cm. The camera was equipped with a tungsten pinhole collimator of 120 mm in focal length and 1.5 mm in diameter for the phantom, 2 mm in diameter for the mouse study. A more complete description of the device, as well as results on small animal imaging can be found in [9]. Prior to reconstruction, we used a correction of the center of rotation applied on the projections after measurement of the defect on a single-line-source phantom as described in [10]. Both the FA-SART and SART algorithms were block iterative with each block corresponding to a different image of projection.

### 3.2. Simulation

The simulation performed here on a computed phantom illustrates the typical behavior of the FA-SART algorithm, which accelerates the convergence when the PSF is large (see Figures 4(a), 4(b) and 4(e), 4(f)). For a thin PSF, no visible difference appears since FA-SART acts on the deconvolution. Bigger structures should not be reconstructed with $\rho$ close to 1 from noisy data to avoid having the high frequencies of the noise emphasized. Three iterations were computed for all reconstructions.

### 3.3. Phantom

A phantom, seen in Figure 5, with three types of cylindrical cavities (diameters 1 mm, 1.5 mm and 2 mm separated with the same distance), was filled with $^{99m}$Tc, 20 MBq activity. The parameters for the acquisition were 30 mm radius of rotation, 60 projections of $64 \times 64$ pixels on 180° and 1 minute per projection. For the comparison in Figures 5(b)–5(d), we used the FA-SART algorithm with $\rho = 1$ and the SART algorithm (which is FA-SART with $\rho = 0$). For this highly detailed object, the FA-SART reconstruction (5(b)) does better than 3 iterations of SART (5(c)) or even 10 iterations (5(d)), due to an important smoothing effect during the first iterations. Figure 5(e) provides a promising way to use FA-SART in a preconditioning of system (1). It shows a reconstruction obtained after 3 iterations of FA-SART ($\rho = 1$) followed by 2 iterations of SART.

### 3.4. Cardiac mouse imaging

For mouse heart perfusion, a normal adult female CD1 mouse (Mouse Clinical Institute, Ilkirch, France) weighting 30 g was injected with 400 MBq of 99mTc-Tetrofosmin (Amersham, General Electric Healthcare, USA). The radius of rotation of the camera was 2.5 cm corresponding to a zoom factor of 5 and 48 projections of $64 \times 64$ pixels were acquired over 180° (see [9] for details). Figure 6 demonstrates a satisfying result with FA-SART and $\rho = 0.8$ that makes both the left and right ventricles visible.

### 4. DISCUSSION

From the obtained results, it seems that the FA-SART algorithm is able to reconstruct high details in less iterations than SART. While the reconstructions performed from physical structures (phantom and animal) show a faster convergence to what seems to be the true distribution, those performed from simulated data demonstrate that this acceleration increase with the PSF's width of the system, that is, when the intrinsic resolution decreases. These simulations also prove that the improvement obtained with the FA-SART algorithm

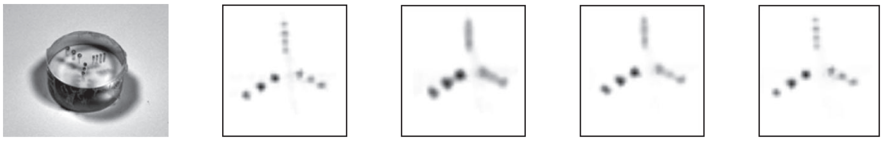

(a) (b) (c) (d) (e)

Figure 5: Photo of the phantom with 3 types of cavities (diameters 1 mm, 1.5 mm, and 2 mm separated by the same distance) which can be filled with a radioactive tracer (a). Transaxial slices of the reconstructed phantom with 3 iterations of FA-SART and $\rho = 1$ (b), 3 iterations of SART (c), and 10 iterations of SART (d). Figure (a) shows significant improvements even when compared with 10 iterations of SART. Figure (e) shows the result obtained from an interesting combination of the two algorithms: 3 iterations of FA-SART ($\rho = 1$) followed by 2 iterations of SART.

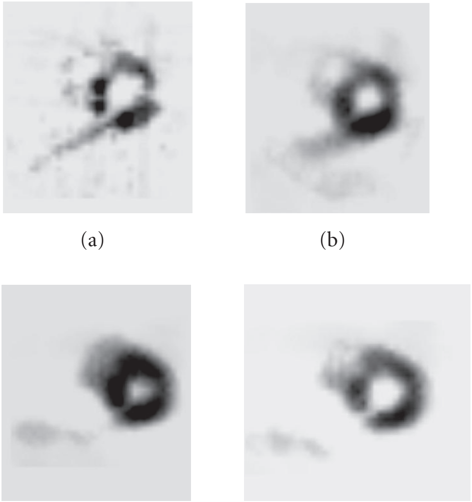

(a) (b) (c) (d)

Figure 6: Normal mouse heart perfusion. Reconstruction with 3 iterations of FA-SART, $\rho = 1$ (a), 3 iterations of FA-SART, $\rho = 0.8$ (b), 3 iterations of SART (c), and 10 iterations of SART (d).

is not due to an inadequacy of the matrix $A$ that models the system. Thus, the method only applies to a class of problems with large PSF, that is, involving an important step of deconvolution like SPECT or optical tomography. It would make no sense, for those applications where the PSF is close to a Dirac function like in computed tomography scanning, to contract the backprojection cone since this cone is already almost a line. It should also be recalled that the gain obtained on highly detailed structures is balanced by a loss in large and homogeneous structures, for which the smoothing properties of the SART algorithm reduce the noise. On the contrary, using the FA-SART algorithm with a high threshold $\rho$ would increase the noise by emphasizing the high frequencies in the reconstruction.

This leads to the major difficulty of such an algorithm: how it should be used. We have shown results with a same value for $\rho$ for all the voxels and all the iterations. This is a first way to accomodate to the FA-SART: the context of SPECT is that of functional imaging, in which in general only one structure is considered interesting. Thus, even if several structures appear in the same image, the parameter $\rho$ can be set to a value corresponding to a given organ or a type of examination (myocardial perfusion produces a sharper image of the heart than blood-pool imaging so these two explorations of the same organ would require different values of $\rho$). This has been for the most part our way to proceed thus far, although other uses can be considered.

A use in preconditioning of the system (1) with two or three iterations of FA-SART followed by a solving with the SART algorithm seems to combine the best of these two algorithms by providing both a fast reconstruction of the high frequencies and an adequate rendering of the bigger structures. Also a value of $\rho$ adapted to the local frequencies of the projection images might successfully change the local properties of the reconstruction, with a high value of $\rho$ only when high-frequencies are detected. We started to investigate this way by creating local frequencies maps of the projection images composed of wavelets coefficients and this will constitute the base of our further works.

A noticeable positive point of the FA-SART algorithm is that it requires very few adaptations when SART has already been implemented. On a programming point of view, certain loops are just partially executed, which also means that one iteration of FA-SART is slightly faster than one of SART.

Since the backprojection operation significantly differs from other studied algorithms, it is by no means obvious to deduce that the FA-SART algorithm converges from an analysis of SART or of the general Landweber scheme. Results concerning the convergence have nevertheless been greatly improved by the suggestion of an anonymous referee and a paper dedicated to a mathematical analysis will be presented elsewhere for publication. This theoretical work will support the experimental evidences shown here, with a study of the convergence of the algorithm but also of its behavior in function of the main frequency.

### 5. CONCLUSION

The FA-SART algorithm has been designed to be able to change the usual rate of convergence of iterative algorithms. While low frequencies are generally the first reconstructed, we permitted to invert this phenomenon in order to have the details appear in the image after very few iterations. We evidenced this behavior of the FA-SART algorithm by showing applications and proposed several possible ways to use it. Theoretical questions will be discussed in a forthcoming paper.

### ACKNOWLEDGMENTS

This work has been permitted by a very profitable collaboration between the Institute of Advanced Research in Mathematics and the Service of Biophysics and Nuclear Medicine in Strasbourg, thanks to Cyrille Blondet, Eric Sonnendrücker, and Stéphanie Salmon. All of our thanks and acknowledgement to Ariel Bardi from Bard College, NY, USA, for assistance in English.

### REFERENCES

[1] R. Gordon, R. Bender, and G. T. Herman, "Algebraic reconstruction techniques (ART) for three-dimensional electron microscopy and X-ray photography," *Journal of Theoretical Biology*, vol. 29, no. 3, pp. 471–481, 1970.

[2] A. H. Andersen and A. C. Kak, "Simultaneous algebraic reconstruction technique (SART): a superior implementation of the art algorithm," *Ultrasonic Imaging*, vol. 6, no. 1, pp. 81–94, 1984.

[3] A. Dempster, N. Laird, and D. Rubin, "Maximum likelihood from incomplete data via the EM algorithm," *Journal of the Royal Statistical Society, Series B*, vol. 39, no. 1, pp. 1–38, 1977.

[4] Y. Censor and S. A. Zenios, *Parallel Optimization: Theory, Algorithm, and Optimization*, Oxford University Press, New York, NY, USA, 1997.

[5] S. J. Norton, "Iterative reconstruction algorithms: convergence as a function of spatial frequency," *Journal of the Optical Society of America A*, vol. 2, no. 1, pp. 6–13, 1985.

[6] S. D. Metzler, J. E. Bowsher, K. L. Greer, and R. J. Jaszczak, "Analytic determination of the pinhole collimator's point-spread function and RMS resolution with penetration," *IEEE Transactions on Medical Imaging*, vol. 21, no. 8, pp. 878–887, 2002.

[7] M. Jiang and G. Wang, "Convergence studies on iterative algorithms for image reconstruction," *IEEE Transactions on Medical Imaging*, vol. 22, no. 5, pp. 569–579, 2003.

[8] Y. Censor and T. Elfving, "Block-iterative algorithms with diagonally scaled oblique projections for the linear feasibility problem," *SIAM Journal on Matrix Analysis and Applications*, vol. 24, no. 1, pp. 40–58, 2003.

[9] A. Constantinesco, P. Choquet, L. Monassier, V. Israel-Jost, and L. Mertz, "Assessment of left ventricular perfusion, volumes, and motion in mice using pinhole gated SPECT," *Journal of Nuclear Medicine*, vol. 46, no. 6, pp. 1005–1011, 2005.

[10] K. Ogawa, T. Kawade, J. Jolkkonen, K. Nakamura, A. Kubo, and T. Ichihara, "Ultra high resolution pinhole SPECT for small animal study," *IEEE Transactions on Nuclear Science*, vol. 45, no. 6, part 2, pp. 3122–3126, 1998.

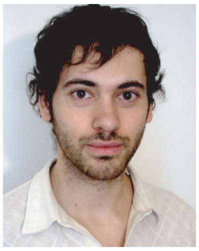

**Vincent Israel-Jost** received his M.S. degree in applied mathematics in 2002 and his Engineer degree from Ecole Nationale Supérieure de Physique de Strasbourg, France. Currently, he is a doctorate student in applied mathematics at the Biophysics and Nuclear Medicine Department of CHU Hautepierre, and the Institute of Research in Advanced Mathematics at Université Louis Pasteur, both in Strasbourg, France. His work concerns the production or improvement of iterative algorithms in cone-beam SPECT. His research interests include iterative algorithms and image processing, as well as philosophy of science and image depiction. He is now pursuing a second Master's degree in the philosophy of science from the University of Paris I - La Sorbonne.

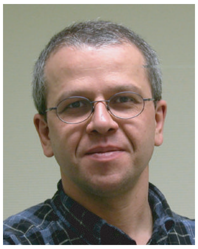

**Philippe Choquet**, veterinary since 1988, got a predoctoral degree in medical imaging and signal processing in 1991, and a Ph.D. degree dissertation in 1996. Since 2001, he has been an Associate Professor in the Biophysics and Nuclear Medicine Department, CHU Hautepierre, Strasbourg. He is the author and coauthor of 29 papers in peer-reviewed journals as well as 76 oral and poster communications in national and international meetings in the field of low field MRI, NMR, and MRI of hyperpolarized gases and small animal scintigraphy.

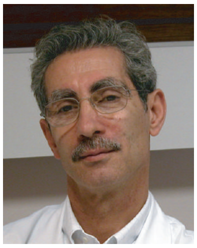

**André Constantinesco** got the Master's degree in physics (1969), Ph.D. degree in physics (1976). He has been an MD since 1977 and a Nuclear Medicine Specialist since 1980 from Université Louis Pasteur of Strasbourg, France. He has been a Professor of biophysics and nuclear medicine since 1981 and Director of the Service de Biophysique et Médecine Nucléaire at Hautepierre University Hospital in Strasbourg since 1997. He is the author and coauthor of 176 publications and 277 oral or poster presentations in the fields of blood haemodynamics, biomechanics, medical instrumentation, nuclear medicine, low field MRI, hyperpolarized xenon MRI, and molecular imaging.

